# Supplementary material for: FMRP expression in primary breast tumor cells correlates with recurrence and specific site of metastasis
Source: PLoS One. 2023 Jun 28;18(6):e0287062. doi: 10.1371/journal.pone.0287062 (PMC10306178; doi:10.1371/journal.pone.0287062)
Supplement: Attachment — Submitted filename: Caredda et al_01_Response to Reviewers.docx [file pone.0287062.s002.docx]

# **Rebuttal letter PONE-S-22-37225 – Caredda et al.**

“Response to reviewers”

Dear Editor,

I am pleased to submit to PLOS ONE, on behalf of all authors, a revised version of the manuscript “*FMRP expression in primary breast tumor cells correlates with recurrence and specific site of metastasis*”, that addresses the points raised during the review process.

I also want to thank you for your interest in this research paper and your kind cooperation.

*Journal Requirements:*

Regarding ethical approval (item 2), we would like to clarify that no participant in our study was under 18 years of age.

We have provided the correct grant numbers for the awards received for our study in the “Funding information” section (item 3).^[1]^

Regarding “data available upon request”, we have uploaded the minimal data set underlying the results of our research to show its features (point 5).

I would also like to thank the reviewers for their important contribution to this study through the development of focal points in the development of the paper.

*The following corrections have been made to the review*:

Concerning the possible molecular mechanism by which FMRP regulates the metastatic event, and the possible role of organ environment in breast cancer the following part was added in the discussion: *“Growing evidence demonstrated that the tumor microenvironment, characterized from different type of cells, such as fibroblasts, endothelial cells, immune cells, etc. and a specific extracellular matrix (ECM) composition, has a crucial role in primary tumor development and metastasis formation (38,39). A worse patient outcome in a metastatic breast cancer is also defined by the presence of specific extracellular matrix proteins and enzymes involved in ECM degradation (40,41). Because several studies reported an alteration of the connective tissues in FXS, highlighting the role of FMRP in maintaining ECM homeostasis (42), it is tempting to hypothesize that FMRP could regulate the composition of tumor microenvironments, affecting the final outcome of metastasis. In addition, FMRP regulates mRNAs encoding for the extracellular matrix proteins, such as integrins and cadherins (26,29,43) as well as matrix metalloproteinases (MMPs), which are involved in the degradation of the ECM (44).*

*Metastasis formation requires several steps including invasion of surrounding tissues, intravasation, survival in the blood circulation, extravasation and colonization/growth in the secondary organ, processes characterized by cellular and molecular peculiar changes (45). FMRP is known to bind and regulate, positively or negatively, the metabolism of several mRNAs that encode for enzymes implicated in extracellular matrix remodeling (46,47,48) therefore FMRP could have a specificity of action in the different organs.”*

To better clarify the FMRP expression in the basal subtype the following part of the discussion was modified: *“We confirmed an increase in FMRP levels in the breast tumor compared to the apparently healthy area surrounding the tumor, consistent with previous findings (29,37). In addition, our analysis revealed that in basal-like tumor subtype, the worst BC associated with the shortest survival time and poor clinical outcome, the high levels of FMRP are independent from the metastatic event. Although the number of samples is low, these data suggest that FMRP is mainly involved in the aggressiveness of this tumor type rather than the metastasis development. In contrast, within the HER2-positive cases, which was the most representative subtype in our cohort, we observed that lower levels of FMRP in the primary tumor were associated with the presence of metastasis.”*

Additional measures of FMRP in the metastatic foci need to be clarified: Only tissue from the primary tumor was available for the study. No analysis of metastatic foci was performed. We may have misunderstood something in the paper. We did not actually analyze the metastatic foci associated with the primary lesions. We analyzed primary tissue and followed patients over time who did or did not develop metastases. Our data are follow-up data, not direct cytological analysis of metastatic tissue.

The legends for table 4 and Figure 2 have been revised and clarified for what they are showing, and we hope that their meaning is now clear.

For Table 4, we confirm that "yes" and "no" mean that metastasis is or is not present in that organ. It should also be noted that the meaning of the FMRP staining refers only to the primary site of the tumor, which may or may not have developed metastases in that organ over time (follow-up).

We confirm that Figure 2 shows an inverse correlation between Cases and Controls, which is unambiguously confirmed by the statistical tests performed. Therefore, the averages between Cases and between Controls are strongly different (statistically significant). In another part of the article, only the behavior of the Cases is described. The variability of FMRP expression within the group of Cases is related to the different sites of metastasis. However, this variability does not overlap with the values of the Controls: the averages between Cases and Controls remain statistically different in an inverse correlation. The red bar is certainly an average, expressing a different FMRP staining according to the metastatic site, but this does not cancel out the differences found with Controls. The two are not mutually exclusive.

Unfortunately, sample size is one of the stated limitations of our work, which is why we were limited in exploring broader perspectives. This did not allow for a complete representation of all molecular subtypes, especially for TNBC.

Apart from our study (and the cited references), there are no other studies correlating FMRP and metastatic BC. There are no preclinical models of this correlation. With this article, we hope to direct research towards the study of such models.

We have replaced Figure 1 with higher quality histological images to provide a more representative and consistent illustration of the results.

A technical clarification: all samples were fixed, processed and immunostained in a standardized manner using automated machines. This eliminates any possible technical issues between samples.

With regard to the additional concerns we have changed/modified the text as suggested by the reviewers.

In conclusion, as mentioned in the discussion, further interdisciplinary research is needed to define the role of FMRP as a prognostic factor for the specific site of metastasis. We hope to further understand the biological mechanism underlying these strong correlations.

Thank you very much for your attention.

Your sincerely,

*Prof Leonardo Palombi*

1. *If you would like to make changes to your financial disclosure, please include your updated statement in your cover letter. (Editor)*
